# Complete Genome Sequence of Staphylococcus aureus CI/BAC/25/13/W, Isolated from Contaminated Platelet Concentrates in England

**DOI:** 10.1128/MRA.00840-21

**Published:** 2021-11-11

**Authors:** Carina Paredes, Sylvia Ighem Chi, Annika Flint, Kelly Weedmark, Carl McDonald, Jennifer Bearne, Sandra Ramirez-Arcos, Franco Pagotto

**Affiliations:** a Centre for Innovation, Canadian Blood Services, Ottawa, Canada; b Department of Biochemistry, Microbiology, and Immunology, University of Ottawa, Ottawa, Canada; c Bureau of Microbial Hazards, Health Products and Food Branch, Health Canada, Ottawa, Canada; d National Health Service Blood and Transplant, London, United Kingdom; University of Arizona

## Abstract

We present the genome sequence of Staphylococcus aureus CI/BAC/25/13/W, which was isolated in 2013 as a contaminant of a platelet concentrate with abnormal clotting at the National Health Service Blood and Transplant. Assessment of the genome sequence showed the presence of one chromosome (2,719,347 bp) and one plasmid (1,533 bp).

## ANNOUNCEMENT

Staphylococcus aureus is naturally present in the mucosa of healthy humans, and it is responsible for community- and health care-associated infections (1, 2). A major platelet concentrate (PC) contaminant (3), it is introduced into donated blood during venipuncture and is predominant in PCs due to the growth-promoting storage conditions for this blood product (4). S. aureus often escapes detection during routine PC screening with culture methods, sometimes causing septic transfusion reactions (3, 5).

Here, we announce the whole-genome sequence of S. aureus strain CI/BAC/25/13/W, part of hemovigilance studies in the United Kingdom. PC samples isolated by the National Health Service Blood and Transplant (NHSBT) from a 5-day-old contaminated split apheresis PC unit were cultured in the BacT/Alert system and yielded positive results within 3 h (6). For DNA isolation, S. aureus CI/BAC/25/13/W was streaked on blood agar plates from frozen stocks at −80°C, and single colonies were cultured at 35°C in 5 ml Trypticase soy broth with 0.6% yeast extract (7). Cells were collected by centrifugation and resuspended in DNA/RNA Shield tubes (Cedarlane), and DNA was extracted using the Quick-DNA high-molecular-weight (HMW) MagBead kit (Zymo Research Corp.) with lysozyme and RNAse A treatment according to the manufacturer’s manual. The same DNA extraction was used for both Nanopore and Illumina libraries.

Paired-end Illumina sequencing was performed using the Nextera XT DNA library preparation kit and a MiSeq instrument (v3 chemistry, 2 × 300-bp reads; Illumina Inc.) according to the manufacturer’s instructions. Nanopore sequencing libraries were constructed using the rapid barcoding sequencing kit (SQK-RBK004) and run using a FLO-MIN106 flow cell (R9.4) and a 1D MinION system (Oxford Nanopore Technologies) for 16 h according to the manufacturer’s protocol. Signal processing, base calling, demultiplexing, and adapter trimming were performed using Guppy (Guppy GPU v3.3.3+fa743ab).

Illumina reads (987,908 reads) were processed using fastp v0.20.0 (8) to remove adapter and barcode sequences, to correct mismatched bases in overlaps, and to filter low-quality reads, resulting in 971,430 filtered reads. From the Nanopore data (28,406 raw reads), reads of <1 kb were removed using Filtlong v0.2.0 (https://github.com/rrwick/Filtlong), resulting in 21,886 filtered reads with an *N*_50_ value of 11,786 bp. Hybrid assembly using Illumina and Nanopore filtered reads was performed using Unicycler v0.4.8 (cluster, reconcile, partition, and consensus functions, with default circularization and rotation) (9) in normal mode, yielding a closed, circular genome comprising a 2,719,347-bp chromosome and a circular 1,533-bp plasmid, with a GC content of 32.88% and average coverage of 96.2× and 61.5× for Illumina and Nanopore data, respectively. Genome annotation was performed using PGAP (release 2020-09-24.build4894; best-placed reference protein set) with GeneMarkS-2+ (https://github.com/ncbi/pgap) and analyzed with QUAST v5.0.2 (https://github.com/ablab/quast) (10). Default parameters were used for computational tools except where otherwise noted.

A total of 2,699 features were identified, including 2,537 genes, 79 pseudogenes, 0 CRISPR arrays, 19 rRNAs, 60 tRNAs, and 4 noncoding RNAs. PC storage conditions facilitate S. aureus proliferation and virulence enhancement, posing a serious health risk. S. aureus CI/BAC/25/13/W was assigned to sequence type 12 (ST12) based on the PubMLST database (11).

### Data availability.

This genome is available in GenBank and the Sequence Read Archive (SRA) under the accession numbers indicated in Table 1.

**TABLE 1:** Provenance and NCBI accession numbers for the CI/BAC/25/13/W isolate

Parameter	Details
Isolate	CI/BAC/25/13/W
BioProject accession no.	PRJNA703973
GenBank accession no.	
Chromosome	CP071102
Plasmid	CP071103
SRA accession no.	
Illumina reads	SRR13745244
Nanopore reads	SRR13745250
Country (region)	United Kingdom (England)
Year	2013
